# Dual-Function Au@Y_2_O_3_:Eu^3+^ Smart Film for Enhanced Power Conversion Efficiency and Long-Term Stability of Perovskite Solar Cells

**DOI:** 10.1038/s41598-017-07218-4

**Published:** 2017-07-28

**Authors:** Chang Woo Kim, Tae Young Eom, In Seok Yang, Byung Su Kim, Wan In Lee, Yong Soo Kang, Young Soo Kang

**Affiliations:** 1 Korea Center for Artificial Photosynthesis and Department of Chemistry, Sogang University, #1 Shinsu-dong, Mapo-gu, Seoul, 121-742 Republic of Korea; 2 Department of Graphic Arts Information Engineering, College of Engineering, Pukyong National University, 365, Sinseon-ro, Nam-gu, Busan, 48547 Republic of Korea; 3 Department of Chemistry and Chemical Engineering, Inha University, Incheon, 402-751 Republic of Korea; 4 Center for Next Generation Dye-Sensitized Solar Cells and WCU, Department of Energy Engineering, Hanyang University, Seoul, 133-791 Republic of Korea

## Abstract

In the present study, a dual-functional smart film combining the effects of wavelength conversion and amplification of the converted wave by the localized surface plasmon resonance has been investigated for a perovskite solar cell. This dual-functional film, composed of Au nanoparticles coated on the surface of Y_2_O_3_:Eu^3+^ phosphor (Au@Y_2_O_3_:Eu^3+^) nanoparticle monolayer, enhances the solar energy conversion efficiency to electrical energy and long-term stability of photovoltaic cells. Coupling between the Y_2_O_3_:Eu^3+^ phosphor monolayer and ultraviolet solar light induces the latter to be converted into visible light with a quantum yield above 80%. Concurrently, the Au nanoparticle monolayer on the phosphor nanoparticle monolayer amplifies the converted visible light by up to 170%. This synergy leads to an increased solar light energy conversion efficiency of perovskite solar cells. Simultaneously, the dual-function film suppresses the photodegradation of perovskite by UV light, resulting in long-term stability. Introducing the hybrid smart Au@Y_2_O_3_:Eu^3+^ film in perovskite solar cells increases their overall solar-to-electrical energy conversion efficiency to 16.1% and enhances long-term stability, as compared to the value of 15.2% for standard perovskite solar cells. The synergism between the wavelength conversion effect of the phosphor nanoparticle monolayer and the wave amplification by the localized surface plasmon resonance of the Au nanoparticle monolayer in a perovskite solar cell is comparatively investigated, providing a viable strategy of broadening the solar spectrum utilization.

## Introduction

Till the perovskite solar cells using an organometallic halide light absorber since a nanocrystalline TiO_2_ film with an organic sensitizer, the perovskite solar cells have been recognized as the most prospective energy generators^1–3^. Pioneering studies of Grätzel *et al*. and the Snaith group on high power conversion efficiencies (PCEs) in perovskite solar cells demonstrated that a mechanism involving successive photon absorption and charge transport leads to record-high PCEs^3–7^. This research has resulted in the fabrication of a promising perovskite solar cell, while using spiro-OMETAD as a hole transfer material (introduced by Prof. N-G. Park) resulted in a PCE of 17%, while perovskite solar cells fabricated by Seok *et al*. reached a PCE of 17.9%. These results have deepened our understanding of the systematic components inside perovskite solar cells and have guided research efforts to improve photovoltaic performance^8, 9^.

The first use of methylammonium halide perovskite featuring an AMX_3_ structure (A = Cs, CH_3_NH_3_; M = Sn, Pb; X = halide) as a three-dimensional (D-π-A) sensitizer for dye-sensitized solar cells (DSSCs) was reported by Miyasaka *et al*. in 2009^10^. Two years later, the newly developed solid-state perovskite solar cell by Park *et al*. spurred considerable interest in the photovoltaic field^6, 11, 12^ with its high performance originating from the high light absorption and a long charge diffusion path provided by the halide-based perovskite structure^13–16^. Recently, several strategies, such as the design and development of perovskite structures, have been successfully demonstrated for the enhancement of solar light conversion efficiency in perovskite solar cells. However, considering the standard AM 1.5 G sunlight spectrum, the visible light range of 280 to 800 nm has been utilized in perovskite solar cells. Additionally, their long-term stability has been considered another critical issue. The PCE enhancement and instability to moisture and UV light are still problems that need to be solved^17, 18^. Considering the drawback of perovskite degradation in perovskite solar cells, a significant enhancement of long-term stability, such as thermal and moisture stability, should be achieved for the commercialization of these solar cells^14, 19^.

A promising approach for utilizing the complete AM 1.5 G spectrum without any wavelength loss is the introduction of phosphors into perovskite solar cells. Despite being considered a promising solution to improve a light harvesting efficiency, the utilization of phosphors in perovskite solar cells has not been extensively researched to date. From this viewpoint, our work suggests a synergetic strategy for enhancing the PCE and stability of perovskite solar cells using a dual-function smart film composed of an Au nanoparticle monolayer on an Y_2_O_3_:Eu^3+^ down-conversion phosphor nanoparticle monolayer. To date, out of a large number of strategies for PCE enhancement in perovskite solar cells, few have been successful^20, 21^. In this aspect, we have recently reported highly qualified phosphor materials^22^ for wavelength conversion, showing that their utilization in DSSCs increases the extent of photon absorption by organic dyes^23^. We have successfully demonstrated that phosphor-induced wavelength conversion of solar light is a promising solution for improving light harvesting efficiency, in agreement with theoretical predictions^23, 24^. In the present work, to the best of our knowledge, we present a first time report of amplified wavelength conversion using both localized surface plasmon resonance (LSPR) and a down-conversion phosphor for the utilization of unused UV light for the enhancement of photovoltaic efficiency and durability of perovskite solar cells. Au nanoparticles on the phosphor layer amplify the converted visible light, increasing its absorbed quantity. Additionally, the hybrid smart film improves the stability of perovskite solar cells by suppressing the degradation of perovskite by converting UV light into visible light during irradiation. This work describes the most viable strategy for not only utilizing the complete solar light spectrum with the help of wavelength conversion, but also achieving a long-term stability of perovskite solar cells.

## Results and Discussion

**Figure 1:**
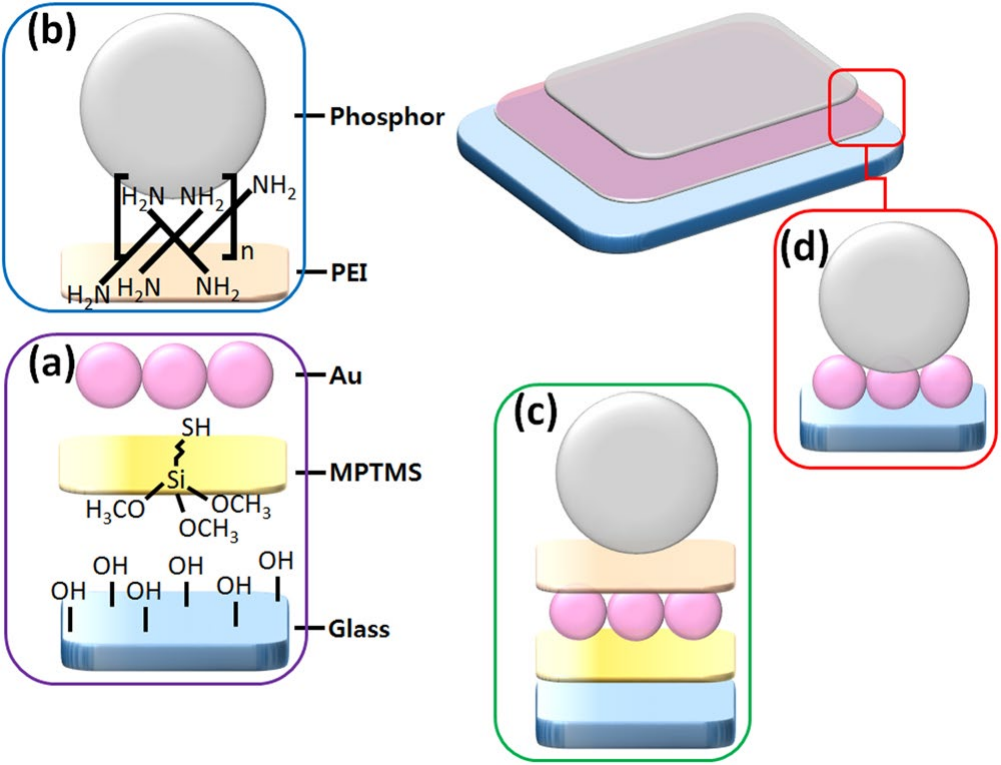
Schematic illustration of a dual functional hybrid film. Formation of Au monolayer on substrate with MPTMS (**a**), phosphor monolayer by polymer induced manual assembly (**b**), phosphor-stacked Au monolayer on substrate (**c**) and Au@phosphor dual function hybrid film (**d**).

**Figure 2:**
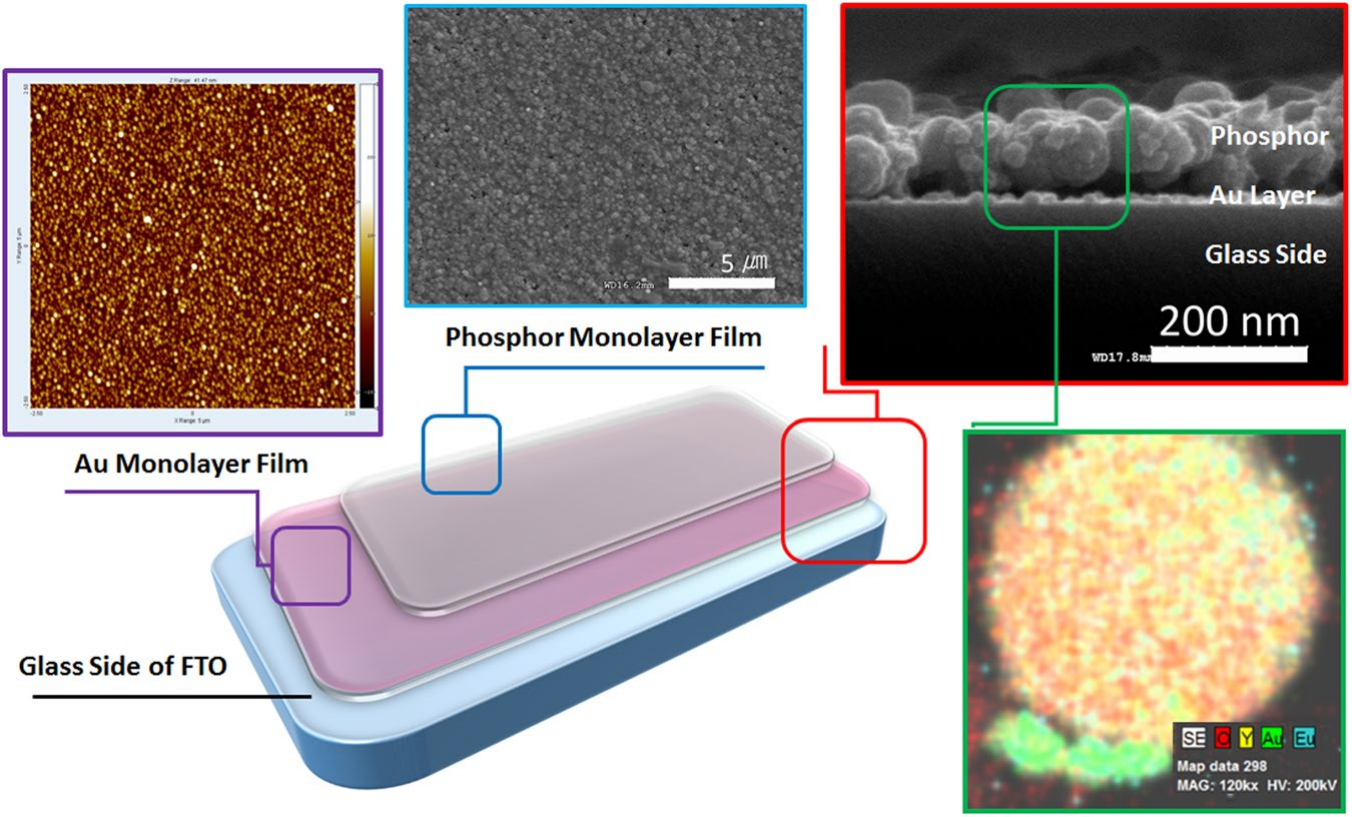
Microscopic observation of a dual functional film. AFM image of Au monolayer film (purple square), Top-viewed (blue square), cross sectional viewed (red square) SEM image and TEM-EDS mapping image (green square, 100 nm × 100 nm) of phosphor monolayer on Au monolayer.

The dual-function smart film was fabricated on pre-synthesized functional nanoparticles using a layer-stacking approach consisting of the following steps: 1) chemical synthesis of type-1 functional nanoparticles, 2) production of a self-assembled monolayer film of functional nanoparticles, and 3) stacking of type-2 functional nanoparticles via polymer-induced manual assembly^25, 26^, as shown in Fig. 1. Representative Au nanoparticles with diameters of ca. ~10 nm (based on TEM imaging results) as type-1 functional materials were synthesized via the two-phase Brust method^27^ using tetraoctylammonium bromide (TOAB) (Figure S1). To prepare the Au monolayer film, the glass side of an FTO substrate was first surface-treated with (3-mercaptopropyl) trimethoxysilane (MPTMS). The glass surface was thiol-functionalized using adhesion between the methoxy groups of MPTMS and the activated hydroxyl groups on the glass surface, as shown in Fig. 1(a). As such, Au nanoparticles were covalent bonded with the thiol groups of MPTMS and were monolayered on the substrate surface after its immersion into the Au nanoparticle sol. Subsequent heat treatment successfully produced the Au monolayer film on the glass surface of the FTO substrate, as revealed by the cross-sectional SEM image in Fig. 2 and the AFM image in Figure S2, which show a homogeneous surface coverage by the self-assembled Au monolayer with uniform thickness.

Y_2_O_3_:Eu^3+^ spherical nanoparticles were synthesized as type-2 functional material using CTAB as a size control agent^28^ and their microscopic structure was characterized using HR-TEM as shown in Figure S3. Typical TEM images and the SAED pattern indicate that the as-synthesized phosphor comprised spherical nanoparticles with ~100 nm diameter. HRTEM imaging proved the exact lattice position, and the lattice parameter was determined as 0.307 nm, precisely matching the (222) plane of bixbyite Y_2_O_3_:Eu^3+^ (Figure S4). The XRD patterns of Y_2_O_3_:Eu^3+^ phosphor nanocrystals and thin film are shown in Figure S5. The diffraction peaks of Y_2_O_3_:Eu^3+^ nanoparticles were matched to the cubic phase of pristine Y_2_O_3_ (PDF# 41–1105). Substitution of Y^3+^ sites in the Y_2_O_3_ host lattice by Eu^3+^ ions caused the peak positions of the Y_2_O_3_:Eu^3+^ phosphor particles to be slightly shifted to lower 2θ values compared to those of the pristine cubic-phase Y_2_O_3_ crystals^22, 28^.

**Figure 3:**
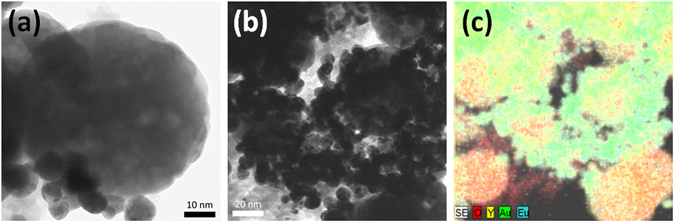
TEM and EDS observation of a dual functional film. Typical TEM images (**a**,**b**) and EDS mapping image (**c**) of phosphor monolayer on Au monolayer. EDS mapping image (**c**) is obtained from TEM image (**b**).

**Figure 4:**
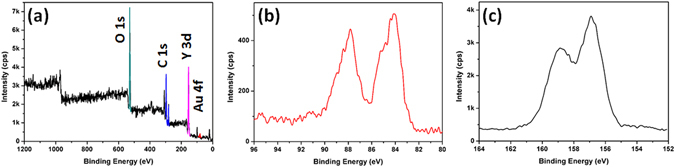
XPS spectrum. Typical XPS spectrum (**a**), Au 4f_7/2_ (**b**) and Y 3d (**c**) of hybrid dual-layer film.

The monolayer phosphor film on the Au monolayer (Au@Y_2_O_3_:Eu^3+^ layer) was fabricated by polyethylenimine (PEI)-induced manual assembly^25, 26^. The imine groups of PEI formed covalent bonds with the oxygen atoms of phosphors on the Au surface, resulting in a monolayer of phosphor particles on the Au monolayer. As shown in Fig. 1 (b), the phosphor nanocrystals were assembled into a monolayer thin film by finger-rubbing phosphor particles on the Au monolayer. The SEM images of the fabricated phosphor monolayer in Figs 1c,d and 2 indicate that the spherical Y_2_O_3_:Eu^3+^ nanocrystals were very uniformly coated on the Au monolayer film. Due to annealing at 450 °C, the phosphor nanoparticles strongly adhered to the substrate during the degradation of PEI, as shown in Figs 1d and 2. Notably, each monolayer was stacked as a hybrid-dual layer, as evidenced by cross-sectional SEM imaging with energy dispersive X-ray spectroscopy (EDX) mapping imaging (Fig. 2). TEM images and the EDX mapping image in Fig. 3 show that the Au@Y_2_O_3_:Eu^3+^ hybrid double-layered thin film composed of the Y_2_O_3_:Eu^3+^ nanoparticle layer and Au monolayers featured stable bonding of the phosphor nanocrystals with Au particles after thermal treatment at 450 °C. XPS chemical state analysis of the Au@Y_2_O_3_:Eu^3+^ hybrid film (Fig. 4) shows that the peak at 84.0 eV was assigned to Au 4f_7/2_, while those at 156.7 and 158.7 eV were exactly consistent with the 3d_5/2_ and 3d_3/2_ transitions of Y_2_O_3_, respectively^29, 30^.

**Figure 5:**
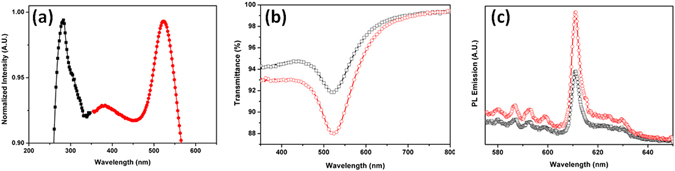
Optical spectrum. Typical UV-vis spectrum (**a**), transmittance (**b**) and PL spectrum (**c**). Phosphor (black) and Au (red) in UV-vis spectrum (**a**). Phosphor monolayer (black) and hybrid dual layer (red) in transmittance (**b**). Phosphor monolayer (black) and hybrid dual layer (red) in PL spectrum (**c**).

To investigate the optical properties of the hybrid dual layer film, the Au nanoparticle and phosphor nanoparticle monolayer films were comparatively studied by UV-vis and PL spectroscopy, as shown in Fig. 5. The localized surface plasmon absorption peak of the Au nanoparticle monolayer film was detected in the visible region at ~520 nm^31^, while the phosphor particle layer showed an absorption peak at ~290 nm, as displayed in Fig. 5(a). The transmittances of the Y_2_O_3_:Eu^3+^ nanoparticle monolayer film and the Au@Y_2_O_3_:Eu^3+^ hybrid double-layered thin film showed a negligible difference, despite the additional stacking of the phosphor layer, as shown in Fig. 5(b). The photoluminescence emission spectra in Fig. 5(c) reveal that the as-prepared Y_2_O_3_:Eu^3+^ nanoparticle monolayer film exhibited a broad absorption band with an emission maximum at ~290 nm and a red-emitted wavelength at 611 nm. Notably, the emission intensity of the Au@Y_2_O_3_:Eu^3+^ hybrid thin film was increased by ~70% compared to the value of the pristine phosphor nanoparticle monolayer film (Fig. 5(c)).

Based on Maxwell’s equations using a quasi-static approximation, the electromagnetic field in a localized surface plasmonic nanoparticle is given by Equation (1)^32^:

1 $${E}_{out}(x,y,z)={E}_{0}\widehat{z}-[\frac{({\varepsilon }_{in}-{\varepsilon }_{out})}{({\varepsilon }_{in}+2{\varepsilon }_{out})}]{\rm{\alpha}}^{3}{E}_{0}[\frac{\widehat{z}}{{\gamma }^{3}}-\frac{\widehat{z}}{{\gamma }^{5}}(x\hat{x}+y\widehat{y}+z\widehat{z})]$$

where ε_in_ is the dielectric constant of metal nanoparticles and ε_out_ is the dielectric constant of the external environment. Given the dimensions of spherical metal nanoparticles, Equation (1) can be rewritten as Equation (2)^33^:

2 $${E}_{out}(\gamma )={E}_{0}\widehat{z}-[\frac{({\varepsilon }_{in}-{\varepsilon }_{out})}{({\varepsilon }_{in}+2{\varepsilon }_{out})}]{\rm{\alpha}}^{3}{E}_{0}[\frac{\widehat{z}}{{\gamma }^{3}}-\frac{3z}{{\gamma }^{5}}\gamma ]$$

Considering that ε_in_ strongly depends on wavelength, the electromagnetic field is determined by both the metal particle size and ε_out_. Based on the ε_in_ of Au (2.25), ε_out_ of air (1.00), and the Au nanoparticle size of ~10 nm, the electromagnetic field is theoretically predicted to be enhanced 100-fold. However, the luminescence enhancement depends on the distance between the plasmonic metal and luminescent materials, and is limited to distances below 2 nm, as recently reported by Kagan^34, 35^. Non-radiative energy transfer (−E_0_) from phosphors to Au nanoparticles causes fluorescence quenching (−3.3 E_0_) and results in ca. 70% emission enhancement for a phosphor to Au nanoparticle distance of less than 2 nm, based on the distance-dependent fluorescence shown in Figure S6(a). It is worth noting that the amplified photoluminescence in Fig. 5(c) is induced by the localized electric field of the Au layer in the Au@Y_2_O_3_:Eu^3+^ hybrid film. Similarly to the optical properties of the hybrid double-layered film, the red emission at 611 nm is attributed to transitions of the charge-transfer state at ~290 nm, which is matched with the red spectrum of the conventional Y_2_O_3_:Eu^3+^ phosphor monolayer^36^. These lines correspond to transitions from the excited 5D_0_ level to the 7F_J_ (J = 0, 1, 2, 3, 4) levels of the Eu^3+^ ion (Figure S6(b)). The most intense line at 611 nm corresponds to the hypersensitive transition between the 5D_0_ and 7F_2_ levels of the Eu^3+^ ion. In addition to its optical properties, the Au@Y_2_O_3_:Eu^3+^ hybrid smart film was proven to be very effective for the wavelength conversion and amplification of solar light, enhancing the performance of perovskite solar cells, as shown in Figure S6(b). Coupling between the LSPR of the Au monolayer and the visible light emitted by the down-conversion phosphor nanocrystal monolayer leads to enhanced efficiencies of perovskite solar cells.

**Figure 6:**
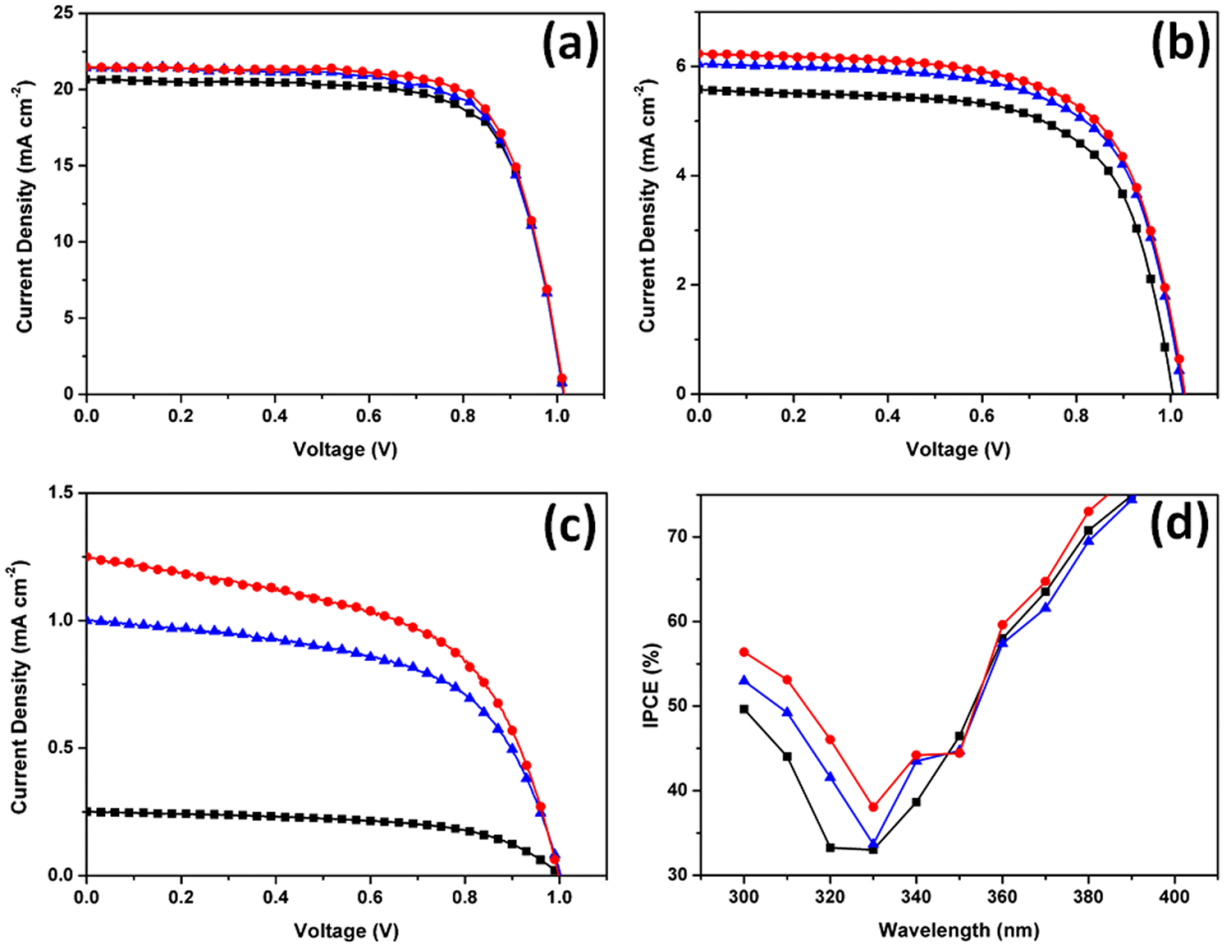
Photovoltaic performance. The comparative photovoltaic performances of standard perovskite solar cell (black), with phosphor monolayer film (blue) and with Au@Y_2_O_3_:Eu^3+^ dual-hybrid film (red). I-V curve under 1-sun (**a**), below 400 nm (**b**) and below 300 nm of wavelength (**c**), and IPCE (**d**).

The increased photocurrent density curves attributed to down-conversion and LSPR are shown in Fig. 6, which compares the photovoltaic performances of a standard perovskite solar cell (ST), a perovskite solar cell with a phosphor monolayer film (PH), and a perovskite solar cell with a Au@Y_2_O_3_:Eu^3+^ dual-functional film (DF) under 1-sun illumination (Fig. 6(a)) and at wavelengths below 400 nm (Fig. 6(b)) and 300 nm (Fig. 6(c)). The short circuit current values (J_sc_) of ST and PH solar cells were determined as 20.7 and 21.4 mA cm^−2^, respectively, under 1-sun illumination. The open circuit voltages (V_oc_) and fill factors (FFs) of ST and DF solar cells were almost the same. The difference of Jsc between ST and PH solar cells was caused by the wavelength conversion effect of the phosphor monolayer film. Notably, the J_sc_ of the PH solar cell was increased despite the slightly decreased optical transmittance. The J_sc_ of the DF solar cell was measured as 21.5 mA cm^−2^, considering the fact that UV light cannot be absorbed by the ST solar cell. This indicates that a portion of UV light was converted into visible light by the red-emitting Y_2_O_3_:Eu^3+^ phosphor film under 1-sun illumination. By absorbing more visible light due to the wavelength conversion effect of the Y_2_O_3_:Eu^3+^ phosphor monolayer, the perovskite solar cell can generate more electron/hole pairs.

The enhanced visible light absorption was reflected in an enhanced performance of the perovskite solar cell and increased J_sc_. This photovoltaic performance undergoes a dramatic change as the solar light illumination wavelength range is narrowed to below 300 nm^37^. The J_sc_ values of ST and PH solar cells under UV light illumination were determined as 5.6 and 6.0 mA cm^−2^, respectively, as shown in Fig. 6(b). The corresponding value of the DF solar cell equaled 6.6 mA cm^−2^, being enhanced by 17.9% below 400 nm and wavelength conversion. Strikingly, the DF solar cell showed a J_sc_ value of 1.25 mA cm^−2^ at wavelengths below 300 nm, which was enhanced by 400 and 25% compared to the values of 0.25 and 1.0 mA cm^−2^ exhibited by ST and PH solar cells, respectively (Fig. 6(c)). As expected, this interesting photovoltaic performance originated from the synergetic coupling of wavelength conversion and the LSPR effect of the hybrid double layer. Initially, UV light was converted into visible light by the phosphor monolayer film with a calculated quantum yield of 80%^22^. Subsequently, the produced visible light was amplified by the LSPR of the Au monolayer to achieve a 170% conversion efficiency. Such Jsc enhancement arises from both the down-conversion effect and LSPR. Since more converted and amplified visible light was absorbed by the DF solar cell, larger numbers of electrons and holes were generated. Even though the hybrid double-layer film slightly reduces the transmittance due to reflection and scattering of incident solar light, it enhances visible light absorption owing to wavelength conversion.

Strikingly, the solar cell utilizing a typical Y_2_O_3_ nanoparticle monolayer film without Eu^3+^ showed a decrease of J_sc_ from 20.7 to 19.6 mA cm^−2^ compared to the ST solar cell, as shown in Figure S7 and Table S1. This suggests that the Y_2_O_3_ nanoparticle monolayer film without Eu^3+^ doping does not exhibit the effect of wavelength conversion based on energy diagram in Figure S6(b). Based on the above photovoltaic performance comparison, some significant observations can be made. First, the DF solar cell showed an increased Jsc compared to that of the PH solar cell, while V_oc_ and FF values remained almost unchanged. Second, no synergetic effect was observed when the positions of the Au nanoparticle and phosphor nanoparticle monolayers were changed, indicating that the dense Au nanoparticle monolayer at the bottom generates the localized electric field that allows the down-conversion luminescence intensity to be enhanced. Based on these factors, it is clear that the enhanced photocurrent density in the DF solar cell arises from the synergetic effect of wavelength conversion and LSPR by overcoming the loss of transmittance in the visible range. Detailed photovoltaic performance results are shown in Table S2.

The IPCE spectra of ST, PH, and DF solar cells also proved the increase of J_sc_ and light harvesting efficiency, as shown in Fig. 6(d). Interestingly, the UV light harvesting efficiency of the DF solar cell was higher than those of other cells, as shown in Fig. 6(d). The IPCE of the DF solar cell was increased by 12 and 6% on average between wavelengths of 300 and 325 nm, as compared with the values of ST and PH solar cells, respectively. The maximum external quantum efficiency of the DF solar cell was determined to be as high as 93% (Figure S8). In addition to the increase of J_sc_, the Au@Y_2_O_3_:Eu^3+^ film can increase the stability of the perovskite solar cell by suppressing its exposure to UV light, as shown in Figs S6 and 7.

**Figure 7:**
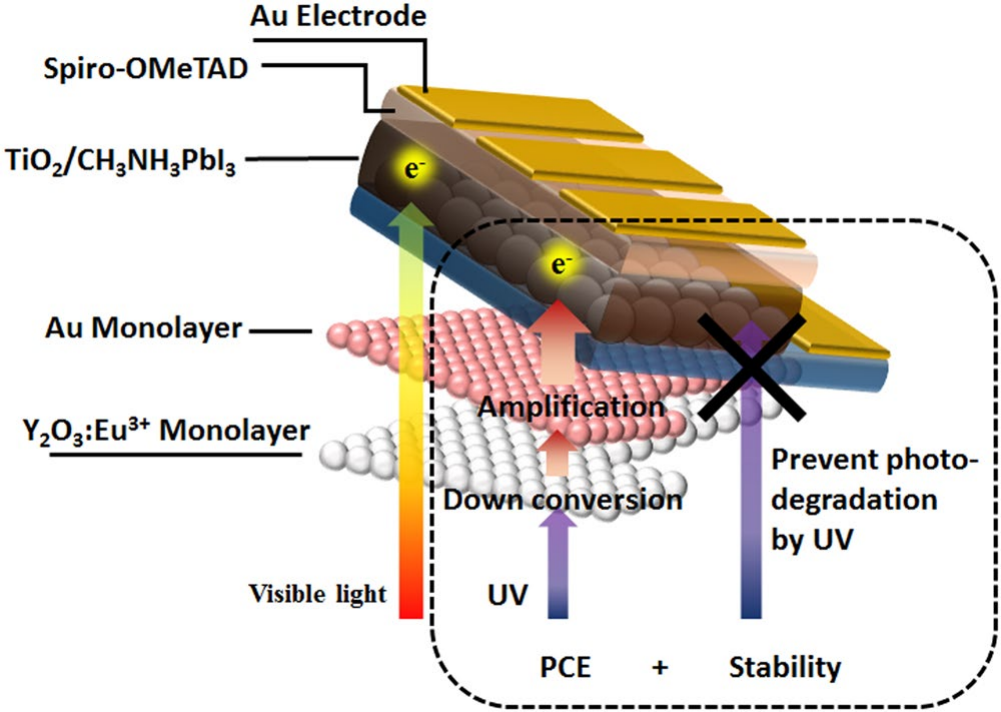
Scheme of dual function smart film. Schematic illustration of dual function Au@Y_2_O_3_:Eu^3+^ hybrid smart film of the perovskite solar cell.

Considering the photodegradation caused by UV light irradiation, the chemical process in the perovskite solar cell can be expressed as follows^14, 19^:

3 $$2{{\rm{I}}}^{-}\leftrightarrow {{\rm{I}}}_{2}+2{{\rm{e}}}^{-}\,({\rm{At}}\,\,{\rm{the}}\,\,{\rm{interface}}\,\,{\rm{between}}\,\,{{\rm{TiO}}}_{2}\,{\rm{and}}\,\,{{\rm{CH}}}_{3}{{\rm{NH}}}_{3}{{\rm{PbI}}}_{3})$$

4 $$3{{\rm{CH}}}_{3}{{\rm{NH}}}_{3}+\leftrightarrow 3{{\rm{H}}}^{+}+3{{\rm{CH}}}_{3}{{\rm{NH}}}_{2}\uparrow $$

5 $${{\rm{I}}}_{2}+{{\rm{I}}}^{-}+3{{\rm{H}}}^{+}+2{{\rm{e}}}^{-}\to 3{\rm{HI}}\uparrow $$

**Figure 8:**
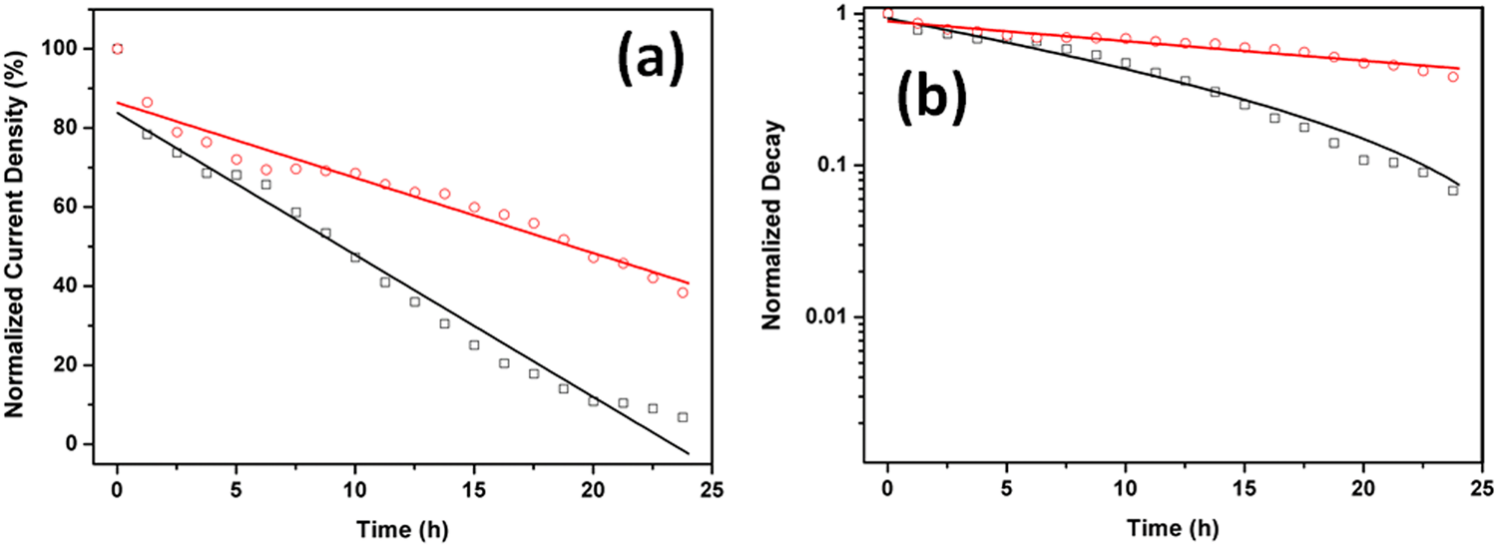
Long-term stability. Comparison of long-term stability (**a**) and time-dependent exponential decay curves (**b**) under UV light illumination between standard perovskite solar cell (black) and with Au@Y_2_O_3_:Eu^3+^ dual-hybrid film (red).

Being located on the front side of the perovskite solar cell, the Au@Y_2_O_3_:Eu^3+^ film can convert UV light into visible light while effectively suppressing the photodegradation caused by UV light exposure. The stabilities of ST and DF solar cells were compared based on their time-dependent photocurrent values, as shown in Fig. 8. Figure 8(a) shows a significant stability difference between ST and DF solar cells. The plot of normalized photocurrent values vs. UV light irradiation time displays the time-dependent stability of the perovskite solar cell related to the photo-degradation of CH_3_NH_3_PbI_3_. As shown by the results of the long-term stability measurement, the photocurrent decreases of ST and DF solar cells show a similar trend for up to 5 h since the start of irradiation due to the degradation of the perovskite surface by both moisture and UV light. After 5 h, the photocurrent of the ST solar cell shows a rapid drop. Due to the ongoing degradation by UV light, the normalized current of the ST solar cell decreases to only 0.1 in 24 h. On the other hand, the DF solar cell shows a slow normalized photocurrent decrease to 0.4 after measuring for 24 h. This comparatively slow photocurrent decay might be explained by the suppression of UV light irradiation, suppressing the photodegradation of perovskite. The rate of photocurrent density decrease of the DF solar cell is relatively slow compared with that of the ST solar cell. The exponential time-dependent fitting curves used are shown in Fig. 8(b), with each fitting value based on the corresponding J_sc_ values of ST and DF solar cells. As shown in Fig. 8(b), the ST solar cell showed a more rapid photocurrent decay compared to the DF solar cell, described by exponential decay (Equation (6))^38^:

6 $${\rm{N}}({\rm{t}})={{\rm{N}}}_{0}\cdot {{\rm{e}}}^{-{\rm{kt}}}$$

Based on the exponential time-dependent curves shown in Fig. 8(b), we calculated the corresponding decay constants (k) related to the degradation of CH_3_NH_3_PbI_3_ under exposure to UV light and moisture. The decay constants for the ST (k_ref_) and DF solar cells (k_DF_) were determined as 25.34 and 16.45, respectively. It is worth noting that the difference between ST and DF solar cells originated from the suppression of UV light irradiation due to the conversion of UV light into visible light.

In conclusion, we demonstrated a dual-function smart film exhibiting the effects of wavelength conversion and a localized surface plasmon resonance for perovskite solar cells. The Au@Y_2_O_3_:Eu^3+^ hybrid smart film was composed of Au nanoparticle and Y_2_O_3_:Eu^3+^ phosphor nanoparticle monolayers, enhancing the power conversion efficiency and long-term stability of the photovoltaic cell. The increase of photocurrent density from 20.7 mA cm^−2^ for the standard perovskite solar cell to 21.5 mA cm^−2^ for the Au@Y_2_O_3_:Eu^3+^ hybrid thin-film solar cell was accompanied by increased stability. This work represents the most viable strategy for not only achieving the utilization of the whole solar spectrum to generate electricity but also for fabricating fortified photovoltaic solar cells.

## Methods

### Materials

Chloroauric acid trihydrate (HAuCl_4_·3H_2_O, 99.9%, Aldrich), tetraoctylammonium bromide (C_32_H_68_BrN, TOAB, 98.0%, Aldrich), and sodium borohydride (NaBH_4_, 99.9%, Aldrich) were used to prepare Au nanoparticles. Sulfuric acid (H_2_SO_4_, 95.0%, Daejung) and hydrogen peroxide (H_2_O_2_, 3.00%, Daejung) were used to prepare the piranha solution for cleaning the FTO substrate. (3-Mercaptopropyl) trimethoxysilane (C_6_H_16_O_3_SSi, MPTMS, 95.0%, Aldrich) was used to fabricate the Au monolayer. Yttrium (III) nitrate tetrahydrate (Y(NO_3_)_3_·4H_2_O, 99.99%, Aldrich) was used as an yttrium source, and europium(III) nitrate pentahydrate (Eu(NO_3_)_3_·5H_2_O, 99.9%, Aldrich) was used as an activator^22^. Cetyltrimethylammonium bromide (C_19_H_42_BrN, CTAB, 99.0%, Aldrich) and urea were used as capping agents. Polyethylenimine (PEI, branched C_2_H_5_N, typical Mw = 25,000; Aldrich) was used for the manual assembly^25, 26^. Ethanol and 2-propanol (C_2_H_6_O and C_3_H_8_O, respectively, 97.0%, Daejung) were used as solvents for cleaning and washing. Ultrahigh-purity deionized water (>18 MΩ, Millipore) was used throughout the experiment. All chemicals were used without further purification.

### Fabrication of Self-Assembled Au Nanoparticle Monolayer Films

In a typical synthetic procedure^27^, 30 mL of 30 mM HAuCl_4_·3H_2_O were added to 80 mL of 50 mM TOAB in toluene under stirring. After the color of the organic layer changed to red, 25 mL of 0.5 M NaBH_4_ were added, and the stirring was continued for 2 h. The resulting reddish-black solution of Au nanoparticles in toluene was washed with 0.1 M H_2_SO_4_ and 1.0 M sodium carbonate aqueous solutions. To prepare the Au nanoparticle monolayer, the FTO substrate was surface-treated with hot piranha solution at 110 °C for 3–5 min. The substrate was dipped into a mixture containing 100 mL of 2-propanol, 5 mL of deionized water, and 5 mL of MPTMS for 10 min, followed by drying in an oven at 106 °C for 10 min. This process was repeated twice to form a thiol layer on the glass surface. Finally, the thiol-functionalized FTO substrate was dipped into a mixture of 30 mL toluene and 1 mL of colloidal Au nanoparticle suspension. After 24 h, the substrate was removed, and its FTO side was blown dry with nitrogen and annealed at 250 °C for 1 h to remove the MPTMS layer.

### Preparation of Wavelength Conversion Layer on Au Nanoparticle Monolayer (Au@Y_2_O_3_:Eu^3+^)

Down-conversion phosphors were synthesized via a modified urea precipitation method followed by thermal treatment^22^. In a typical procedure, 4 mmol of Y(NO_3_)_3_·4H_2_O and CTAB (each) and 80 mmol of urea were added to 100 mL of deionized water. The concentration of Eu(NO_3_)_3_·5H_2_O was fixed to 1 mol.%. The mixed solution was stirred at 80 °C for 2 h under ultrasonication. The obtained white precipitate was washed three times with deionized water and ethanol and dried in an oven at 80 °C for 24 h, followed by annealing in a furnace at 1000 °C for 1 h. To prepare the dual-layer film, 1.0 mL of 1.5 wt.% PEI solution in ethanol was spin-coated on a 2 × 2 cm area of Au nanoparticle monolayer film at 2500 rpm for 30 s. The as-prepared Y_2_O_3_:Eu^3+^ nanoparticles were spread on the PEI-coated Au nanoparticle monolayer film and smoothly finger-rubbed. The manually assembled film was annealed at 450 °C for 4 h to remove the residual PEI.

### Fabrication of Perovskite Solar Cells

The FTO glass substrate (Pilikington, TEC 7) was delicately cleaned using 5.0 wt.% Helmanex solution, deionized water, and ethanol (20 min for each step). To prepare the TiO_2_ compact layer, a ca. 10 nm thick Ti film was deposited on the patterned FTO glass by RF magnetron sputtering (A-Tech system, Korea), followed by oxidation at 500 °C for 30 min in air. A mesoporous TiO_2_ layer approximately 180 nm thick was then spin-coated at 5000 rpm for 30 s, using TiO_2_ pastes containing 20–30 nm size nanoparticles. The coated films were then heated at 500 °C for 30 min. A CH_3_NH_3_PbI_3_ layer was deposited by a previously reported two-step method^9^. A solution of PbI_2_ in N,N-dimethylformamide (462 mg/mL) at 70 °C was spin-coated onto the porous TiO_2_ films at 6000 rpm for 60 s and dried at 70 °C for 30 min. The film was immersed in a CH_3_NH_3_I/2-propanol solution (10 mg/mL) for 20 s and washed with 2-propanol, followed by drying at 70 °C for 15 min. The hole transfer material (HTM) layer was subsequently spin-coated at 4000 rpm for 30 s using a solution containing 72.3 mg of spiro-OMETAD [2,29,7,79-tetrakis(N,N-di-p-methoxyphenylamine)-9,9-spirobifluorene] in 1 mL of chlorobenzene, 28.8 μL of 4-tert-butylpyridine and 17.5 μL of lithium bis(trifluoromethylsulphonyl) imide in 1 mL of acetonitrile, and 29 μL of tris(2-(1-H-pyrazol-1-yl)-4-tert-butylpyridine) cobalt(III) bis(trifluoromethylsulphonyl) imide in 1 mL of acetonitrile. A 60 nm thick Au layer was deposited using a thermal evaporator (Korea Vacuum Tech.) to form the back contact. The device active area was defined by a metal mask with an area of 0.122 cm^2^.

### Characterization and Photovoltaic Performance Measurement

XRD (Rigaku MiniFlex II Desktop X-ray) characterization of the Y_2_O_3_:Eu^3+^ phosphor was performed at 30 kV and 15 mA with Cu Kα radiation (λ = 1.54056 Å). A scanning rate of 0.02° step^−1^ and a 2θ range of 20–60° were used. Imaging of Y_2_O_3_:Eu^3+^ phosphor nanoparticles and their films was conducted using scanning electron microscopy (SEM, cold field emission scanning electron microscope JEOL S-4300) and trans-mission electron microscopy (TEM, JEOL, JEM-2100F, operated at 200 keV). To determine the atomic binding structures and states in the dual-layered film, the binding energies of Au and Y ions were measured by X-ray photoelectron spectroscopy (XPS, K-alpha, Thermo UK) employing a monochromated Al Kα X-ray source. The optical absorption and transmittance of the thin film were determined using a Varian Cary 5000 UV-vis NIR spectrophotometer (Agilent Technologies). Photoluminescence (PL) emission spectra were obtained using a Hitachi F-700 fluorescence spectrophotometer. Additionally, surface imaging of the Au monolayer was per-formed using atomic force microscopy (AFM, S.I.S. Surface Imaging Systems GmbH, Germany) in non-contact mode. P-V measurements were performed using a Keithley model 2400 source measurement unit. A 300-W xenon lamp (Spectra-Physics) was used as the light source, and the light intensity was adjusted using an NREL-calibrated Si solar cell equipped with a KG-5 filter to approximate the AM 1.5 G one-sun intensity. Incident photon-to-current efficiency (IPCE) spectra were recorded for wavelengths from 300 to 800 nm using a specially designed IPCE system (PV Measurements, Inc.) The long-term stability and decay curves were obtained by measuring photocurrent as a function of solar light irradiation time using a PL-9 potentiostat and a simulated solar illumination source (AM 1.5 G filter, Asahi HAL-320, 100 mW cm^−2^). XUS0300 and XUS0400 short-pass filters (Asahi) were used to limit the illumination to wavelengths below 300 and 400 nm, respectively. The decay curves were constructed using the IVIUM CompactStat software, with their slopes indicating the relative stability of solar cells.

## Electronic supplementary material

Supporting Information
