# Supplementary material for: Dual-Function Au@Y2O3:Eu3+ Smart Film for Enhanced Power Conversion Efficiency and Long-Term Stability of Perovskite Solar Cells
Source: Sci Rep. 2017 Jul 28;7:6849. doi: 10.1038/s41598-017-07218-4 (PMC5533740; doi:10.1038/s41598-017-07218-4)
Supplement: Supplementary file 1 — Supporting Information [file 41598_2017_7218_MOESM1_ESM.doc]

Supporting Information

Dual-Function Au@Y_2_O_3_:Eu^3+^ Smart Film for Enhanced Power Conversion Efficiency and Long-Term Stability of Perovskite Solar Cells

Chang Woo Kim, Tae Young Eom, In Seok Yang, Byung Su Kim, Wan In Lee, Yong Soo Kang, and Young Soo Kang*


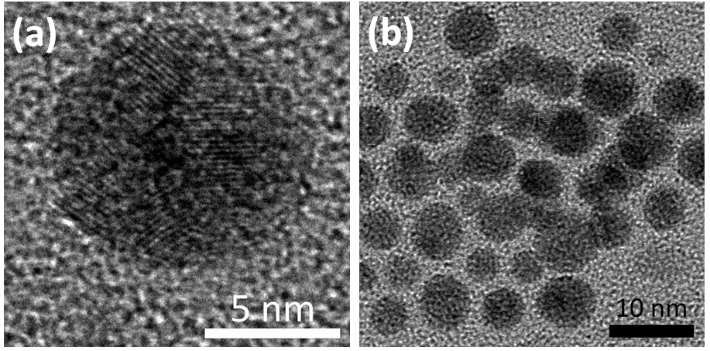


Figure S1. Typical TEM images of as-prepared Au nanoparticles.


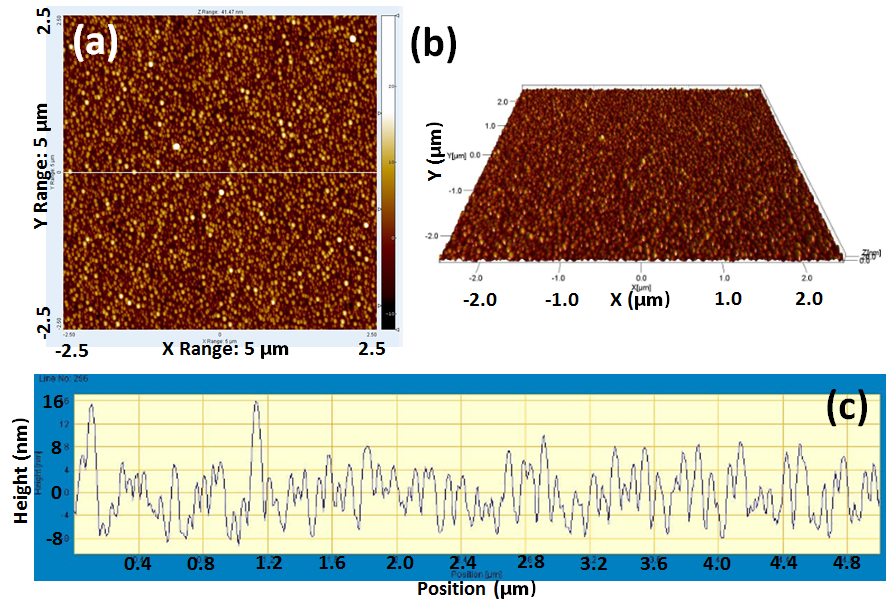


Figure S2. AFM images (a, b) and topographic image (c) of Au nanoparticle monolayer film on glass substrate. Au nanoparticle monolayer by self-assembled adsorption between MPTMS and Au nanoparticles has the uniform thickness.


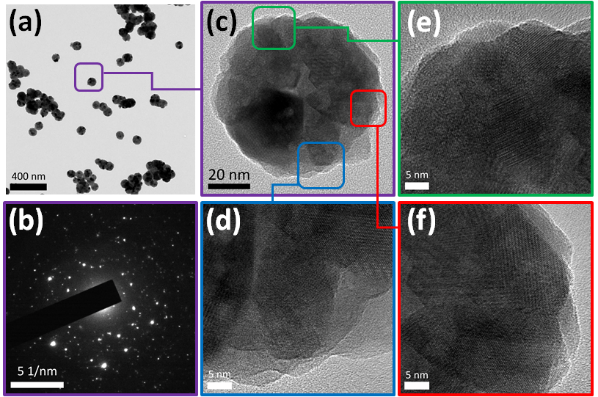


Figure S3. Typical TEM images (a, c-f) and SAED pattern (b) of as-synthesized phosphor nanoparticles. Low resolution TEM image (a) and high resolution TEM images (c, d, e and f).


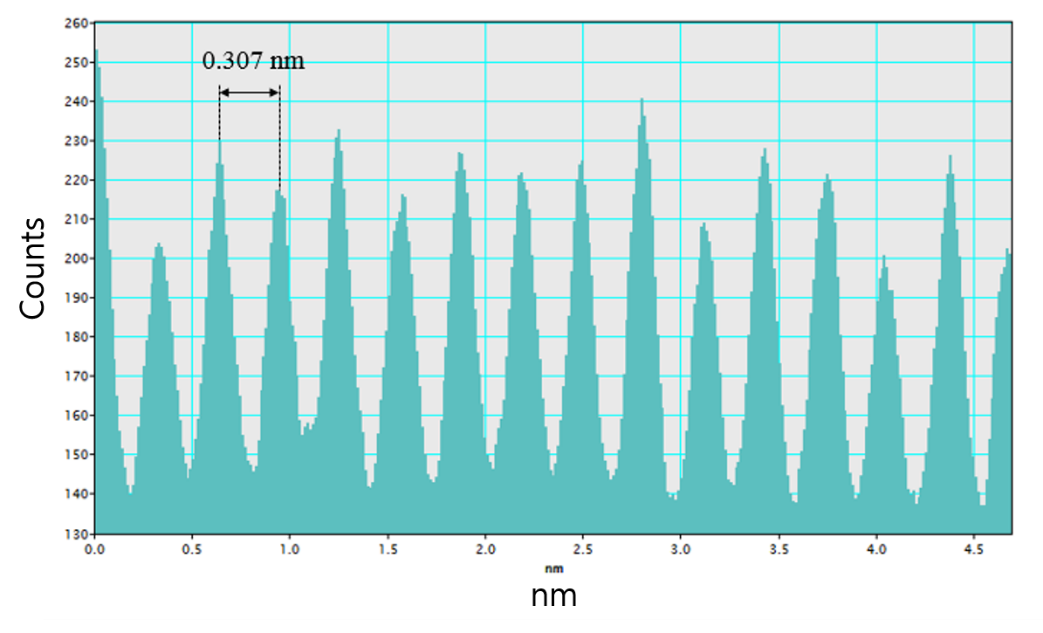


Figure S4. Lattice analysis of the Y_2_O_3_:Eu^3+^ nanoparticles in Figure S3 (c). The distance between two parallel planes was determined as 0.307 nm using Gatan digital micrograph that was installed to TEM. The distance is same as (222) plane of bixbyite Y_2_O_3_ crystal.


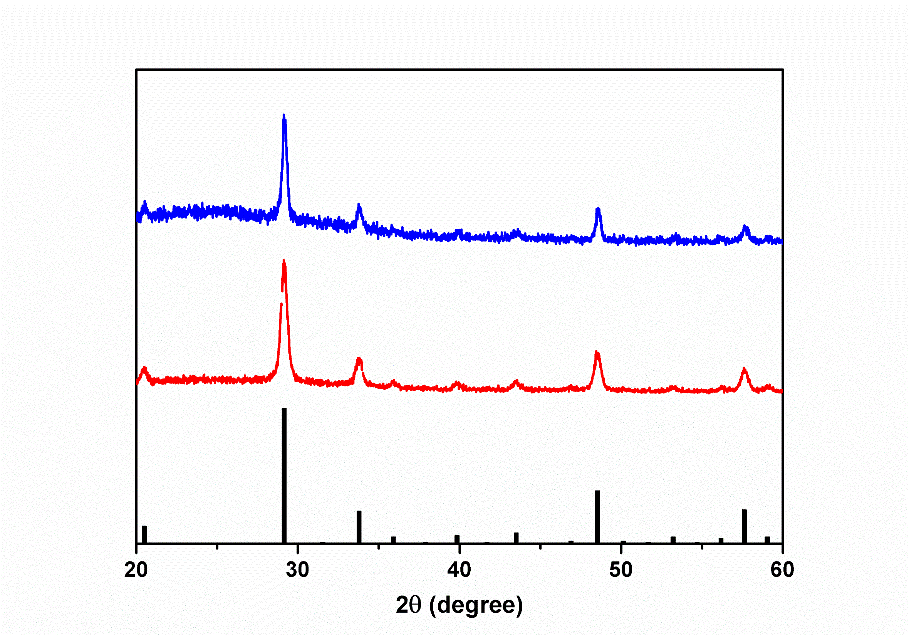


Figure S5. XRD spectra of the Y_2_O_3_:Eu^3+^ nanoparticles (blue), monolayer film (red) and standard Y_2_O_3_ PDF# 41-1105.


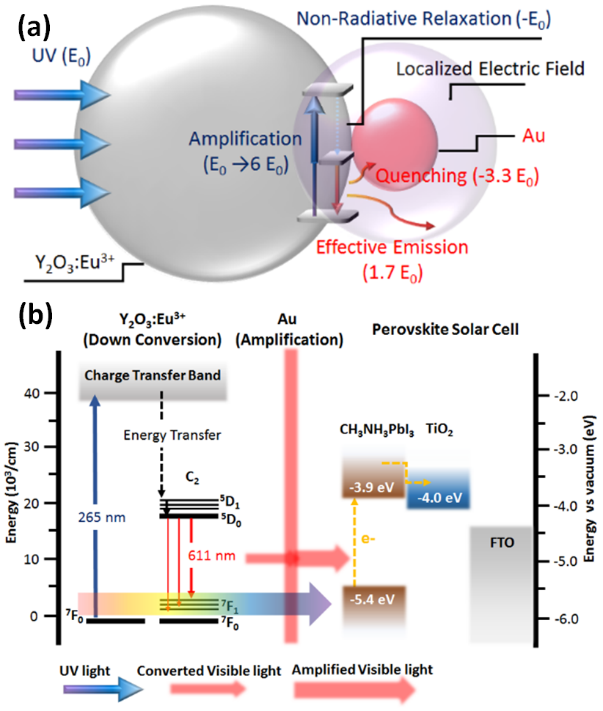


Figure S6. Mechanism of dual function effect. Schematic illustration of LSPR (a) and energy transfer mechanism (b) in Au@Y_2_O_3_:Eu^3+^ hybrid thin film for the perovskite solar cell.


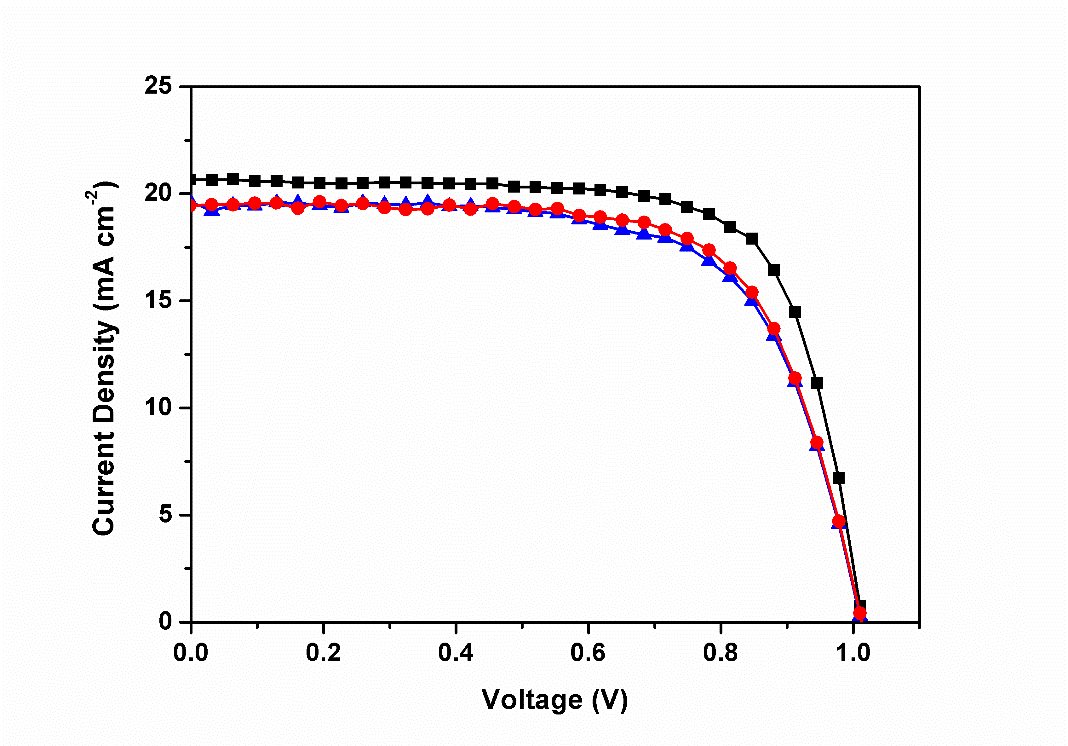


Figure S7. I-V curves of standard perovskite solar cell (black), with Y_2_O_3_ nanoparticle monolayer film (blue) and with Au@Y_2_O_3_ film (red) under 1-sun illumination.

Table S1. Photovoltaic performance data of standard perovskite (ST) solar cell and dual function (DF) solar cells composed of Y_2_O_3_ nanoparticle monolayer film and with Au@Y_2_O_3_ film. A measurement was carried out five times and the average values are shown.

|  | J_sc_  (mA cm^-2^) | V_oc_  (mV) | FF  (%) | Ƞ  (%) |
| --- | --- | --- | --- | --- |
| ST ^a)^ | 20.7 ± 0.2 | 1013.3 ± 0.1 | 72.37 ± 0.01 | 15.16 |
| ST with Y_2_O_3_ ^a)^ | 19.6 ± 0.3 | 1011.9 ± 0.1 | 66.41 ± 0.01 | 13.19 |
| ST with Au@Y_2_O_3_ ^a)^ | 19.4 ± 0.2 | 1012.8 ± 0.1 | 69.00 ± 0.01 | 13.59 |

Table S2. Photovoltaic performance data of standard perovskite (ST) solar cell, phosphor nanoparticle monolayer film (PH) and with hybrid dual film (DF) solar cells. A measurement was carried out five times and the average values are shown.

|  | J_sc_  (mA cm^-2^) | V_oc_  (mV) | FF  (%) | Ƞ  (%) | ∆Ƞ ^b)^  (%) |
| --- | --- | --- | --- | --- | --- |
| ST ^a)^ | 20.7 ± 0.2 | 1013.3 ± 0.1 | 72.37 ± 0.01 | 15.16 |  |
| PH ^a)^ | 21.4 ± 0.3 | 1013.0 ± 0.1 | 72.02 ± 0.01 | 15.62 | 3.03 |
| DF ^a)^ | 21.5 ± 0.3 | 1014.9 ± 0.1 | 74.14 ± 0.01 | 16.06 | 5.94 |

^a)^denoted in Scheme 1; ^b)^∆Ƞ = (Ƞ - Ƞ_ST_ )/Ƞ_ST_ ☓ 100 %


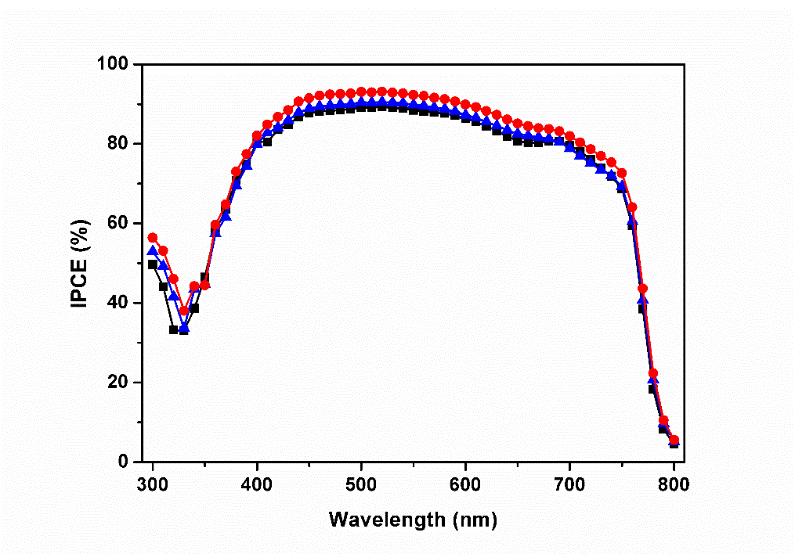


Figure S8. IPCE curves of standard perovskite (black), phosphor nanoparticle monolayer film (blue) and Au@Y_2_O_3_:Eu^3+^ dual-hybrid functional film (red) solar cells.
